# Evidence for cemented TKA and THA based on a comparison of international register data

**DOI:** 10.1007/s00132-024-04489-4

**Published:** 2024-04-03

**Authors:** Martina Humez, Katharina Kötter, Ralf Skripitz, Klaus-Dieter Kühn

**Affiliations:** 1 Institute of Hygiene and Environmental Medicine, Justus-Liebig-Universität Giessen, Schubertstraße 81, 35392 Giessen, Germany; 2 Heraeus Medical GmbH, Wehrheim, Germany; 3 Centre for Endoprosthetics, Foot Surgery, Paediatric and General Orthopaedics, Roland-Klinik Bremen, Bremen, Germany; 4 Department of Orthopaedics and Orthopaedic Surgery, Medical University of Graz, Graz, Germany

**Keywords:** Implant fixation, Bone cement, Periprosthetic fracture, Revision risk, Arthroplasty register, Implantatfixierung, Knochenzement, Periprothetische Frakturen, Revisionsrisiko, Endoprothesenregister

## Abstract

**Background:**

Hip and knee implants can either be fixed without cement, press-fit, or with bone cement. Real-world data from arthroplasty registers, as well as studies provide a broad database for the discussion of cemented versus uncemented arthroplasty procedures.

**Objective:**

What does current evidence from international arthroplasty registries and meta-analyses recommend regarding cemented or cementless fixation of hip and knee implants?

**Methods:**

A recommendation is generated by means of direct data comparison from the arthroplasty registries of eight countries (USA, Germany, Australia, UK, Sweden, Norway, New Zealand, Netherlands), the comparison of 22 review studies and meta-analyses based on registry data, as well as an evaluation of recommendations of healthcare systems from different nations. For this purpose, reviews and meta-analyses were selected where the results were statistically significant, as were the annual reports of the arthroplasty registries that were current at the time of writing.

**Results:**

For knee arthroplasties, long survival time as well as lower risk of revision can be achieved with the support of cemented fixation with antibiotic-loaded bone cement. In patients aged 70 years and older, cemented fixation of hip stem implants significantly reduces risk of intraoperative or postoperative periprosthetic fracture (quadruple). This applies both to elective total hip arthroplasties and to hemiarthroplasty after femoral neck fractures. Antibiotic-loaded bone cement significantly (*p* = 0.041) reduces the risk of periprosthetic infection, especially in patients with femoral neck fractures.

**Conclusion:**

Total knee replacement with antibiotic-loaded bone cement is well established internationally and is evidence-based. Registry data and meta-analyses recommend cemented fixation of the hip stem in older patients. In Germany, USA and Australia these evidence-based recommendations still must be transferred to daily practice.

**Graphic abstract:**

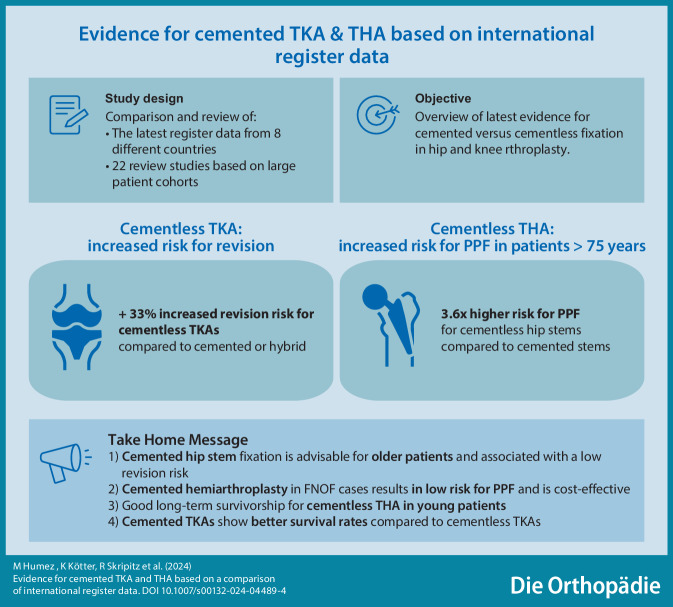

## Abstract (de)

**Hintergrund:**

Hüft- und Knieimplantate können entweder zementfrei, press-fit oder mit Knochenzement befestigt werden. Reale Daten aus Endoprothesenregistern sowie Studien bieten eine breite Datenbasis für die Diskussion zwischen zementierten und unzementierten Endoprothesen.

**Ziel:**

Was empfehlen die aktuellen Erkenntnisse aus internationalen Endoprothesenregistern und Metaanalysen zur zementierten oder zementfreien Fixierung von Hüft- und Knieimplantaten?

**Methoden:**

Mittels direktem Datenvergleich aus den Endoprothesenregistern von acht Ländern (USA, Deutschland, Australien, Großbritannien, Schweden, Norwegen, Neuseeland, Niederlande), dem Vergleich von 22 Übersichtsarbeiten und Metaanalysen auf der Basis von Registerdaten sowie einer Auswertung von Empfehlungen von Gesundheitssystemen aus verschiedenen Nationen wird eine Empfehlung generiert. Hierfür wurden Übersichtsarbeiten und Metaanalysen ausgewählt, deren Ergebnisse statistisch signifikant waren, sowie die Jahresberichte der zum Zeitpunkt der Erstellung der Studie aktuellen Endoprothesenregister.

**Ergebnisse:**

Bei Knieendoprothesen können durch die zementierte Fixierung mit antibiotikahaltigem Knochenzement eine lange Überlebenszeit und ein geringeres Revisionsrisiko erreicht werden. Bei Patienten im Alter von 70 Jahren und älter reduziert die zementierte Fixierung von Hüftschaftimplantaten das Risiko einer intra- oder postoperativen periprothetischen Fraktur signifikant (um das Vierfache). Dies gilt sowohl für elektive Hüfttotalendoprothesen als auch für Hemiarthroplastiken nach Schenkelhalsfrakturen. Antibiotikabelasteter Knochenzement reduziert das Risiko einer periprothetischen Infektion signifikant (p = 0,041), insbesondere bei Patienten mit Schenkelhalsfrakturen.

**Schlussfolgerung:**

Die Knietotalendoprothese mit antibiotikahaltigem Knochenzement ist international gut etabliert und evidenzbasiert. Registerdaten und Metaanalysen empfehlen die zementierte Fixierung des Hüftschaftes bei älteren Patienten. In Deutschland, USA und Australien müssen diese evidenzbasierten Empfehlungen noch in die tägliche Praxis übertragen werden.

For years there has been a trend towards cementless fixation of hip and knee implants in several countries, among them Germany, USA, and Australia. Cemented fixation is sometimes regarded as outdated. Which fixation method is associated with the lowest revision risk and therefore the longest implant survival time? This question is discussed in the following with the aid of data from international arthroplasty registers to achieve an evidence-based conclusion.

Osteoarthritis is the most common reason for arthroplasty, particularly in the knee, hip, ankle, and fingers [29]. Most patients suffering from osteoarthritis are aged 60 years and over, with women being affected significantly more often than men. Fractured neck of femur (FNOF) are the second most common cause of arthroplasty, particularly in the patient cohort aged 80 years and over. The number of intracapsular FNOF increased significantly between 2009 and 2019, with a growth rate of up to 23% [59]. In the age group of 80–89 years, which is most frequently treated with hemiarthroplasty, the annual incidence of FNOF is 884 for women and 569 for men per 100,000 inhabitants in Germany [61]. Based on increased numbers of osteoarthritis and FNOF patients, the number of hip operations increased as well: within the OECD (Organisation for Economic Co-operation and Development) countries, Germany is the leader with 315 hip implants per 100,000 inhabitants in 2019, followed by Switzerland (313), and Austria (295), with a median of the 35 OECD countries being 174 hip implants per 100,000 inhabitants [32, 53]. In comparison, there are significantly fewer arthroplasty procedures in knees: in 2019, 227 were performed in Germany. Here, Switzerland (260 knee procedures per 100,000 inhabitants), Finland (242), and Austria (229) are ahead of Germany, while the average for all 33 OECD countries is 137 knee implants per 100,000 inhabitants [32, 53]. In these frequent knee and hip arthroplasty procedures it is aimed at reducing possible revision risks, as an increased number of primary procedures naturally increases the total number of potential revision procedures. Revisions in general are associated with high costs for hospitals, health insurance systems and negative side effects for patients. An increase in treatment of patients with increased age and patients with multiple comorbidities (ASA [American Society of Anaesthesiologists] score III/IV) was noted. These factors favor an increase of potential revisions. Nevertheless, technical progress has significantly extended long-term survival of implants. According to a study published in the Lancet in 2019, the long-term survival risk for total knee arthroplasty (TKA) is convincing: 93% of TKAs remain unrevised after 15 years, 90% after 20 years, and 82% after 25 years [22]. The survival risk for total hip replacement (THA) is significantly lower with survival risks of 89% after 15 years, 70% after 20 years, and 58% after 25 years [21].

## Aseptic loosening as the most common reason for revision

Currently, the most common reason for revision in TKA, as recorded in the German Arthroplasty Register 2023 (EPRD) [20] and the National Joint Register (NJR) from the UK is aseptic loosening (22.8%, 26.7%, respectively) followed by infection (14.5%, 19.3%, respectively) [47]. In contrast, the Australian Arthroplasty Register (AOANJRR) [5], Dutch Arthroplasty Register (LROI) [17], Swedish Arthroplasty Register (SAR) [39] and the American Joint Replacement Registry (AJRR) [1] highlight infections as the most common reason for revision in TKA and loosening second. According to the EPRD, revision procedures are associated with a high financial burden for hospitals as well as negative health consequences for patients. As a rule of thumb, more than half of all revisions also require complete reimplantation of all primary implant components.

## Cemented TKA as the gold standard

Cemented TKA dominates in Germany: 95.6% of primary TKAs are cemented and a 3.0% are treated using hybrid techniques ([20]; Table 1).

**Table 1:** Share of cemented fixation in primary total knee arthroplasty (TKA) based on Annual Register Reports 2023

	EPRD	AOANJRR	NJR	LROI	SAR	AJRR
	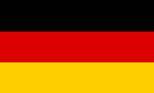	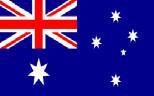	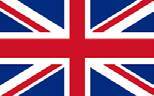	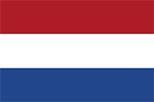	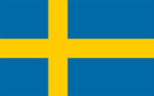	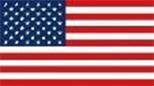
*Cemented (%)*	95.6	61.8	95.5*	92.2	90.6	77.6
*Hybrid (%)*	3.0	18.6	0.8*	2.7	0.5	1.9
*Cementless (%)*	1.3	19.9	3.8*	5.1	8.9	20.5

*Unicondylar knee replacements were excluded for better comparability

*AJRR* American Joint Replacement Registry, *AOANJRR* Australian Orthopaedic Association National Joint Replacement Registry, *EPRD* German Arthroplasty Register, *LROI* Dutch Arthroplasty Register, *NJR* National Joint Registry United Kingdom, *SAR* Swedish Arthroplasty Register

In an international comparison, the LROI [17] and the SAR [39] showed comparable cementation rates over 90% as well as the NJR [47] predominantly using cemented anchoring of TKA 95.5% of the time (Table 1). A slight trend towards cementless procedures has been observed in recent years. The AOANJRR [5] also shows a predominance of cemented replacements in 61.8% of the cases, although the proportion of cementless implants has significantly increased since 2018 and has even reached a share of 19.9% in 2022. This trend is in line with developments in the USA [1], which shows a proportion of 20.5% cementless TKAs in 2022 (Table 1). This trend is supported by new implant developments that are advertised as “modern” on the market. The use of robotics can also be seen as a trend towards the increased use of cementless implants. Similar to the development of the “Robo-Doc”, cementless implants are also used in robotics-assisted operations alongside cemented implants [42].

Ideally, the decision on fixation method should be based on scientific evidence as well as survival rates and failure probabilities. Arthroplasty registers aim to precisely provide this evidence: they were originally set up in Scandinavia to obtain clinically relevant information from pooled data, improve the quality of care, and reduce the number of revision procedures. Thus, arthroplasty registries provide a good overview of reliable implant designs and fixation methods [35]. For cementless TKAs, the EPRD shows a significantly increased probability of failure after 5 years (4.3%) compared to cemented or hybrid fixation (3.6% and 3.8%) (Table 2).

**Table 2:** Failure rate of TKA and THA based on EPRD annual report 2023

Failure rate after …	1 year	2 years	3 years	4 years	5 years	6 years	7 years	8 years
Total knee arthroplasty (TKA)								
Cemented	1.7 (1.6; 1.7)	2.6 (2.5; 2.6)	3.0 (3.0; 3.1)	3.4 (3.3; 3.5)	3.6 (3.6; 3.7)	3.9 (3.8; 4.0)	4.2 (4.0; 4.3)	n/a
Hybrid	1.9 (1.7; 2.1)	2.8 (2.5; 3.0)	3.3 (3.0; 3.6)	3.5 (3.2; 3.8)	3.8 (3.5; 4.1)	4.1 (3.7; 4.5)	4.4 (3.9; 4.9)	n/a
Cementless	2.0 (1.6; 2.5)	3.2 (2.7; 3.9)	3.9 (3.3; 4.7)	4.1 (3.5; 4.9)	4.3 (3.6; 5.1)	4.6 (3.8; 5.5)	4.6 (3.8; 5.5)	n/a
Elective total hip arthroplasty (THA)—hip stem								
Cemented	2.4 (2.3; 2.5)	2.7 (2.6; 2.8)	2.9 (2.8; 3.1)	3.1 (3.0; 3.3)	3.4 (3.2; 3.5)	3.6 (3.4; 3.7)	3.8 (3.6; 4.0)	4.1 (3.8; 4.3)
Cementless	2.7 (2.7; 2.8)	3.2 (3.1; 3.2)	3.4 (3.3; 3.5)	3.6 (3.5; 3.7)	3.8 (3.7; 3.8)	3.9 (3.9; 4.0)	4.1 (4.0; 4.2)	4.3 (4.2; 4.5)

Failure rate of TKA and THA based on EPRD annual report 2023 in % including range

**Fig. 1:**
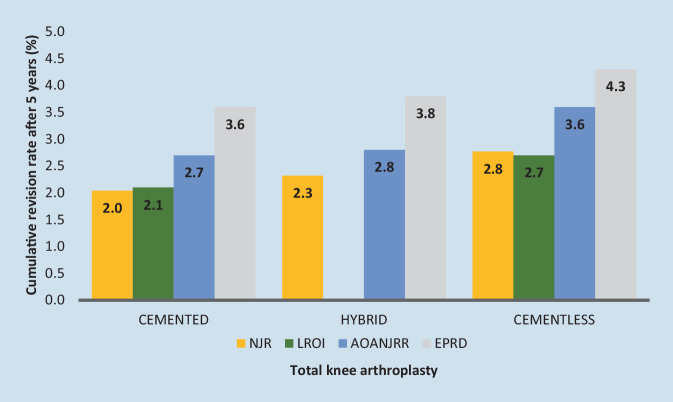
Cumulative revision rate for total knee arthroplasty after 5 years based on register data from the annual reports 2023 from Germany (EPRD), Australia (AOANJRR), UK (NJR) and the Netherlands (LROI)

A comparison of these results with data from other registries leads to the same conclusion: after 5 years, cementless TKA has an increased probability of failure of up to 19% [20] compared to cemented TKA (Fig. 1). This is also supported by the relevant literature: in 2009 Ghandi et al. [25] demonstrated superiority of cemented TKA due to higher survival rates compared to cementless TKA and the associated lower risk of revision for aseptic loosening. The odds ratio (OR) for cementless TKAs was 4.2 (95% confidence interval, CI 2.7–6.5, *p* < 0.0001).

In 2021 Irmola et al. [30] investigated the 10-year survival rate of cementless, cemented, hybrid, and inverse hybrid TKAs based on data from the Nordic Arthroplasty Register Association (265,877 TKAs) resulting in an increased risk of revision of cementless fixation (hazard ratio 1.3, 95% CI 1.1–1.4) compared to cemented fixation. In this study, even after 15 years cemented TKAs show good survival rates in contrast to cementless TKAs presenting most of the failed implants. The cumulative 15-year revision rate of minimally stabilized TKAs is lower with cemented fixation than with cementless, whereby hybrid fixation has the lowest revision rates according to the AOANJRR [4]. The NJR [46] and the New Zealand Arthroplasty Register (NZJR) [62] confirm this finding, in patients aged 65–74 years, in particular. Research has shown that the revision rate in patients over 75 years of age is significantly lower when TKA was cemented compared to cementless or hybrid fixation [30]. The reduced risk of revision associated with cemented fixation may be due to the “forgiving” effect of bone cement: the use of bone cement compensates for possible deficits in placement and prosthesis design [33]. In addition to survival rates, patient satisfaction with the implant is increasingly being recognized. Analyses of the NZJR show no significant differences in short-term and long-term patient satisfaction between the different treatments, although cementless TKAs have higher revision rates than fully cemented TKAs [51].

**Fig. 2:**
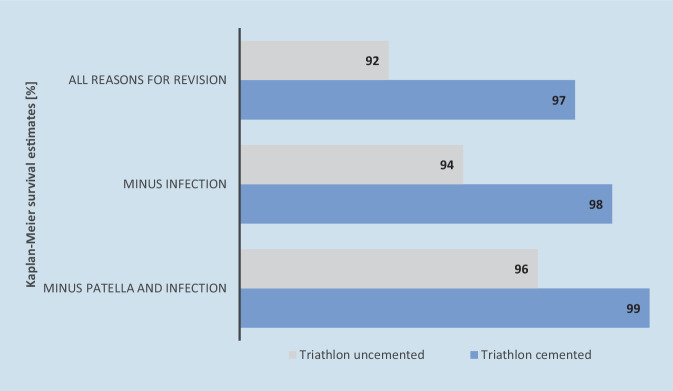
Cumulative revision risk (CRR) with a confidence interval (CI) of 95% for cemented and cementless Triathlon implants. SAR annual report 2022 [63]

The influence of prosthesis design on differences in survival rates of TKAs is currently being discussed. The implant design certainly appears to have an impact on long-term survivorship of knee implants but registry data show that cemented fixation is associated with lower revision risk compared to cementless TKA. An analysis by the SAR [63] showed that in addition to the prosthesis design, the fixation method influences revision risk as well. Simultaneous increases in the proportion of cementless knee prostheses (up to 10%) and in revision risk (up to 6%) prompted the authors of SAR to analyze revision risks for different fixation types. Using the same prosthesis design (triathlon prosthesis, Triatholon Total Knee System, Stryker, Portage, MI, USA), survival rates of cemented and cementless fixation were compared (Fig. 2): cementless TKA achieved cumulative revision risk of 8% compared to cemented TKA at 3% for all reasons for revision. Even when patella replacement and infections are excluded from the reasons for revision, higher cumulative revision risk is shown in cementless fixation (6% and 4%, respectively) than in cemented fixation (2% and 1%, respectively). In addition to low stability of cementless TKA, the experience of orthopedic surgeons in handling cementless implants may play a relevant role [63].

This theory is supported by further studies: in addition to the choice of implant and fixation method, experience of the orthopedic surgeon has decisive impact on survival rates in TKA [28]. Most surgeons who achieved high TKA implant survival rates with their team opted for cemented fixation in combination with implants with highly cross-linked polyethylene. The orthopedic surgeon’s decision for or against a particular combination of fixation method and implant has greater influence on survival rate than the prosthesis design itself [28].

Contrary to the emerging trend towards cementless anchoring, current evidence confirms cemented (hybrid) TKA as the gold standard presenting the lowest risk of revision and highest survival rates as outlined.

## Paradox of cementless hip stems

In Germany, 77% of THAs are performed without cement [20]. In contrast to TKA, the picture is very heterogeneous in other countries as well (Table 3). The LROI [17] shows a trend towards cementless THA (68%) and, in particular, the AJRR [1] (95%) whereas SAR [39] documents a higher proportion of hybrid or cemented THAs with 67% as well as the NJR [47] with 64%.

**Table 3:** Share of cemented fixation in primary total hip replacement based on Annual Register Reports 2023

	EPRD	AOANJRR	NJR*	LROI	SAR	NAR	AJRR
	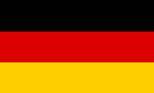	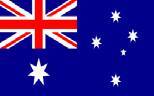	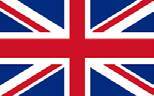	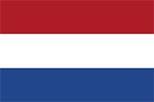	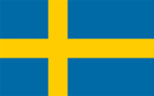	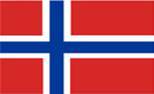	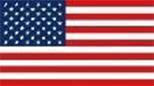
*Cemented (%)*	4	2	19	20	52	18.7	5
*Hybrid (%)*	18	36	40	9	6	25.6	
*Reverse hybrid (%)*	1	n/a	2	3	9	6.9	
*Cementless (%)*	77	62	36	68	33	48.8	95

*Unconfirmed procedures not listed

*AJRR* American Joint Replacement Registry, *AOANJRR* Australian Orthopaedic Association National Joint Replacement Registry, *EPRD* German Arthroplasty Register, *LROI* Dutch Arthroplasty Register, *NAR* Norwegian Arthroplasty Register, *NJR* National Joint Registry United Kingdom, *SAR* Swedish Arthroplasty Register

**Fig. 3:**
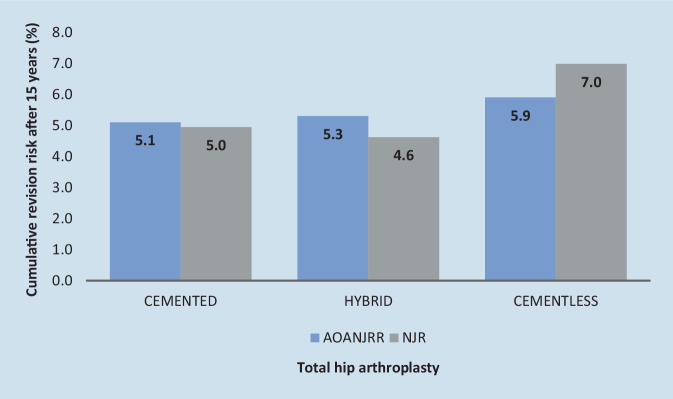
Cumulative revision risk for total hip arthroplasty after 15 years based on register data from the annual register reports 2023 Australia (AOANJRR) and UK (NJR)

What is the difference between cementless and cemented hip stem implants? Cementless fixation requires the press-fit implant growing into the femur in a timely manner, which can be favored by a structured surface of the prosthesis. Only healthy and actively dividing bone tissue can completely enclose the implant and grow onto the surface of the prosthesis. If the patient is affected by osteopenia or osteoporosis, division activity of bone tissue is considerably impaired and complete ingrowth of the implant is substantially complicated [2, 3]. In Europe, it is estimated that 22% of the female population and 7% of the male population over the age of 50 years suffer from osteoporosis [34]. In Germany [19], the patient group aged 75–84 years is most frequently treated with elective THA. Consequently, this patient group, which frequently suffers from the clinical consequences of osteoporosis, is mainly treated with cementless hip stem implants. The EPRD shows increased failure risks for cementless THA (3.8%) after 5 years compared to cemented anchoring (3.4%) (Table 3). At the same time, the AOANJRR and the NJR show lower failure risks for cemented (5.1% and 4.95%, respectively) and hybrid (5.3% and 4.62%, respectively) THAs after 15 years compared to cementless (5.9% and 6.98%, respectively) THAs (Fig. 3). One of the main reasons for hip stem implant failure is the occurrence of intraoperative or postoperative periprosthetic fracture. Both revision due to aseptic loosening and due to a periprosthetic fracture come along with considerable health risk for older patients. The aim should be to reduce all possible revision risks to maintain the quality of life of older patients. The assumption that cementless fixation is associated with an increased risk of revision for older patients is supported by data from the AOANJRR [4]: over the age of 75 years, cementless THA is associated with an increased risk of revision, whereas cemented THA shows the lowest risk of revision. Based on this finding, Babazadeh et al. [6] recommended cemented fixation of the hip stem for both experienced and inexperienced orthopedic surgeons.

In the 2021 annual report the EPRD also posed the question “Is hip stem cementation advisable for older patients” [18]? and concluded that cemented hip stems are advisable for patients over 75 years of age.

**Fig. 4:**
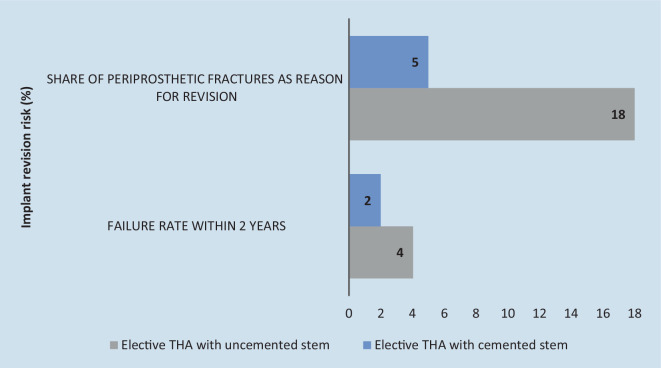
Risk and reason for revision 2 years after surgery for patients aged 75 years and above according to annual report 2021 EPRD

In patients aged 75 years and older the cemented stem fixation showed significantly lower failure risk at 2.0% (1.8%–2.2%) compared to 3.7% (3.4%–4.0%) for cementless stem fixation after statistically matching the comparison groups. The proportion of periprosthetic factures as the reason for revision was significantly higher for cementless stems at 18% compared to cemented stems at only 5% (Fig. 4).

Furthermore, the NAR [50] found significantly better implant survival in women over 75 years when the total hip prosthesis was fully cemented versus uncemented (relative risk, RR 1.59; *p*-value < 0.001).

Recommendations on the choice of fixation method were made by Bunyoz et al. [13] based on a meta-analysis of 10 arthroplasty registries. In patients aged 75 years and older, hip stem implants should be cemented to reduce risk of revision and increase the survival time of the THA. This meta-analysis is consistent with the analysis of 66,955 hip replacements from the Norwegian Arthroplasty Register (2021, NAR) [49]: cementless fixation increases risk of revision due to an increased risk of periprosthetic fractures and dislocation. Cementless hip stem implants show fivefold increased risk (RR 5.2, CI 3.2–8.5) of periprosthetic fractures 90 days after surgery compared to cemented stems [14]. This is a serious risk factor, especially for women over 55 years of age (RR 12, CI 6–25) [14]; however, this also affects young men as an analysis by the NZJR showed [52]. Due to incomplete ingrowth of the cementless implant in combination with increased physical activity, cementless hip stem implants show increased periprosthetic fractures and dislocations in young men. Conversely, this suggests that in older patients with poorer bone quality, possibly due to osteoporosis, implant ingrowth is slower, thus increasing the risk of periprosthetic fractures. Data from the USA, where cementless THA dominates (95%), also showed that cemented anchoring of the hip stem implant is associated with a statistically significant reduction in revisions due to periprosthetic fractures (hazard ratio, HR 0.113, 95% CI 0.052–0.245, *p* < 0.0001) [8]. Springer et al. [60] investigated the paradox of cementless THA in the USA and concluded that a cemented hip stem reduces the risk of early periprosthetic fractures and enables long service life (> 20 years). In Denmark, elective THAs were primarily treated without cement for many years. Based on the evidence in favor of cemented THAs, Denmark decided to change the treatment algorithm: older patients, especially women aged 60 years and over, should be treated with a cemented hip stem implant. Subsequently, Omari et al. [54] analyzed the effects of changed treatment algorithm and documented a significant reduction in periprosthetic fractures (from 4.57% to 1.25%, *p* = 0.007) due to cemented fixation of the femoral implant in women over 60 years of age. The NAR recommends using cemented femoral stems in all women over 75 years of age achieving cementation rate of 87.4% in this patient group [50].

## Cemented hemiarthroplasty for FNOF patients

Comparable to elective THAs the question also arises in the case of a femoral neck fracture: shoul the hip stem be cemented or uncemented? The multicenter randomized controlled trial World Hip Trauma Evaluation 5 (WHiTE 5) analyzed the economic aspects of cemented or cementless treatment after dislocated femoral neck fractures in addition to the revision risk [56]. Not only the actual direct costs for the operation but also the average costs for patient follow-up care over a period of 12 months were considered. Compared with cementless implants, cemented hip stem implants showed a cost saving (£961) with a simultaneous improvement in quality of life (QALY [quality-adjusted life year] survey of patients). The authors therefore concluded that cemented THA is a cost-effective treatment for FNOF patients [51].

The German Society for Orthopedics and Trauma Surgery (DGOU) also recommends the following in its “White paper on geriatric traumatology and orthogeriatrics” [41]: cemented stem implants for patients with FNOF or osteoporotic bone structure. Cementing the femoral component enables better distribution of the load to the bone and thus reduces the risk of periprosthetic fractures. In a direct comparison of FNOF patients who were treated with cemented hip stems (610 patients) or with cementless implants (615 patients), a periprosthetic fracture occurred in 2.1% of cases in the cohort of patients treated without cement, while 0.5% occurred in the group of patients treated with cement. Fernandez et al. (2022) [24] showed that cemented hemiarthroplasty after FNOF results in a lower risk of periprosthetic fractures (0.5% cemented compared to 2.1% cementless) and a significantly better quality of life for patients aged 60 years and above.

## Cemented hip stem fixation is a cost-efficient treatment

As many healthcare systems are under cost pressure, treatment should be cost-effective in the long term. To reduce the expenditure of the British National Health Service (NHS) while maintaining the same quality of life for the patient, an evidence-based treatment recommendation getting it right first time in orthopedics (GIRFT) for hip replacements was made: patients aged 70 years and above should receive a fully cemented or hybrid hip replacement [11]. To reduce the expenditure (AUS $ 2 million over 5 years) of the Australian healthcare system, Blythe et al. [9] recommended to change the treatment algorithm from cementless to cemented for both elective hip replacement and hemiarthroplasty after FNOF. A comprehensive review of current studies on the cost-effectiveness of different fixation methods showed that cemented or hybrid fixation is the most cost-effective choice. Only in very young patients (below the age of 43 years) is a cementless hip stem implant the most cost-effective choice [64].

## Hip stem implant design with low revision risk

**Fig. 5:**
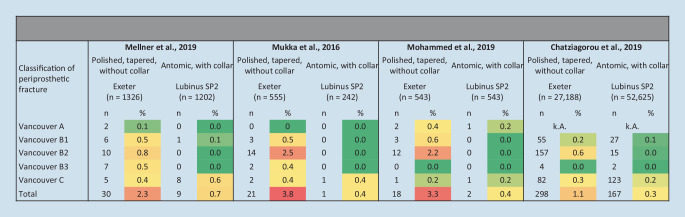
Comparison of the types of periprosthetic fractures after cemented hip stem implantation with an Exeter or Lubinus SP2 stem. Color coding representing the risk for revision as following dark green = 0.0%, light green = 0.1%–0.2%, yellow = 0.3%–0.5%, dark yellow = 0.6%–1.0, light orange = 1.1%–2.0%, dark orange = 2.1%–3.0%, red = 3.1–3.8%

The evidence recommends cemented fixation of the hip stem implant, but which implant design should be chosen? Is a polished tapered stem without collar (e.g., Exeter, Exeter hip stem, Stryker, Portage, MI, USA) or an anatomically shaped implant with collar (e.g. Lubinus SP II, Waldemar Link, Hamburg, Germany) the best choice (Fig. 5)? Implant designs differ in terms of fracture risk: polished tapered stems without collars have higher risks of early periprosthetic fracture compared to anatomically shaped stems with collars [12]. Chatziagorou et al. [26] showed, based on data from the Swedish registry, the influence of implant design on fracture type: risk of Vancouver type B fracture is increased in contrast to type C fractures. This finding indicates the relevance of the hip stem design on the fracture type. Exeter stems have a 10-fold increased risk of type B fracture compared to Lubins SPII stems [26]. This correlation has been studied several times in the literature: cemented, polished tapered stems without collars show an increased incidence (3.8%) of early periprosthetic fractures for patients over 80 years of age compared to cemented, anatomical hip stem implants (0.4%) [45]. Furthermore, Mellner et al. [43] showed an increased risk of periprosthetic fractures for the Exeter implant (2.3%) compared to the Lubinus SPII implant (0.7%) and, therefore, recommend using Exeter implants in FNOF patients with great caution. A polished stem without collar, as opposed to an anatomically formed stem, should be used with caution according to Mohammed et al. [44] as it comes with an increased incidence (3.3%) of periprosthetic fractures especially in FNOF patients. In conclusion, the best treatment for older patients and FNOF patients is cemented fixation with an anatomically shaped hip stem with collar.

## No increased mortality due to cemented fixation

Although the advantages of cemented hip stems are manifold, in some countries (e.g., USA, Germany) orthopedic surgeons decide against cemented stem fixation, presumably due to concerns about possible intraoperative complications. Fat embolisms (also described as bone cement implantation syndrome, BCIS) occur more frequently with cemented procedures than with cementless. When BCIS occurs, a cardiovascular collapse is triggered. This is often associated with an increase in pressure in the femoral canal, which can be observed when the bone cement is pressurized during the cementing process or when the prosthesis is inserted. The BCIS is categorized into 4 different grades (grades 0, 1, 2 and 3) (Table 4); however, the risk of fat embolism can be significantly reduced by consistent use of the third generation cementing technique cleaning the femoral canal with pulse lavage and by reducing the intramedullary pressure [10, 36, 58].

**Table 4:** Classification of the bone cement implantation syndrome (BCIS)

Grade 0	No occurrence of implantation syndrome
Grade 1	Moderate hypoxia (arterial oxygen saturation < 94%) or hypotension (a drop in systolic arterial pressure > 20%)
Grade 2	Severe hypoxia (arterial oxygen saturation < 88%) or hypotension (a drop in systolic arterial pressure > 40%) or unexpected loss of consciousness
Grade 3	Cardiovascular collapse requiring cardiopulmonary resuscitation

Rassir et al. [58] provide a good overview of the incidence of implantation syndrome with their comprehensive meta-analysis of 12 studies: grade 3 only occurs in very rare cases with an incidence of 0.1% of all arthroplasty procedures and mainly affects patients with multiple pre-existing comorbidities (ASA III/IV). The implantation syndrome can occur in arthroplasty procedures on all joints with the highest incidence in cemented hemiarthroplasty (0.4% grade 3) (Table 5). The analysis of 79,557 patients based on data from the NAR showed no difference in mortality for cemented and cementless THAs [15]. The complications observed, both perioperatively and postoperatively, were related to the age and comorbidities of the patients, not to the type of fixation. The incidence of developing BCIS grade 3 was 0.03%. When comparing data from the Australian Hip Fracture Registry with the National Death Index [57] there was no significant association between the use of bone cement and the 30-day mortality rate and 1 year after surgery. Treating FNOF patients with a cemented hip stem is safe and does not increase the risk of mortality for the patient [57]. These findings are confirmed by a systematic review by Dominguez et al. [16] according to which there is no evidence of increased mortality with cemented THA or hemiarthroplasty after FNOF. For each patient, the increased risk of periprosthetic fracture with cementless fixation should therefore be weighed against the manageable risk of fat embolism with cemented fixation.

**Table 5:** Incidence of bone cement implantation syndrome (BCIS) grade 3 for hip arthroplasty

	Ramsay et al. 2023 [57]	Bökeler et al. 2022 [10]	Rassir et al. 2021 [58]	Dale et al. 2020 [15]
Number of patients	*n* = 15,405	*n* = 92	*n* = 3294	*n* = 79,557
Examination period	1 year post-op	3 days post-op	30 days post-op	10 years post-op
ASA classification—patient proportion in %				
ASA 1	2	0	12	20
ASA 2	17	29	60	60
ASA 3	59	67	26	19
ASA 4	22	4	2	0
Age group/average patient age	82 years	84 years	75 years	65–74 years with 35% 75+ years with 29%
*Mortality 3 days post-op in %*	no data available	no data available	no data available	*0.03*
*Incidence BCIS grade 3 in %*	*1.70*	*0.00*	*0.40*	*n/A*

## The impact of bone cement brands on revision risk

Going one step further, the SAR investigated in its annual report 2023 [39] the impact of common bone cement brands on 10-year implant survival in Sweden. In 99,276 hip cups, cumulative revision risks were highest when using Refobacin R (Zimmer Biomet, Warsaw, IN, USA) (CRR 2.4; CI 95%, range 2.3–2.5) and lowest when using CMW 2 (DePuy Synthes, Johnson & Johnson, New Brunswick, NJ, USA) (CRR 2.0; CI 95%, range 1.6–2.5). Adjusted regression analysis results in significantly lower revision risk when using Palacos R+G (Heraeus Medical, Wehrheim, Germany) compared to Refobacin R (RR 0.83; CI 95%, range 0.75–0.92; *p* = 0.001). An analysis of 83,489 cemented hip stems showed higher cumulative risk of revision in hip stems in the Refobacin R group (CRR 2.6; CI 95%, range 2.5–2.7) compared to the Palacos R+G group (CRR 2.1; CI 95%, range 2.0–2.2). In regression analysis, similarly, fixation with Palacos R+G (adjusted RR 0.75; CI 95%, range 0.67–0.85; *p* < 0.001) shows significantly reduced risk ratio compared to Refobacin R (RR 1 reference) after 10 years. In hip prostheses where both cup and stem were cemented with the same cement, in both procedures for any cause (adjusted RR 0.85; CI 95%, range 0.76–0.95; *p* = 0.004) as well as for infections (adjusted RR 0.66; CI 95%, range 0.56–0.78; *p* < 0.001) the Palacos R+G group was associated with significantly lower risk ratios compared to the Refobacin R group. This difference was not found in procedures with other causes than infections. All in all, risk of revision due to infection in hip implants is greater when using Refobacin R compared with Palacos R+G and trend-wise compared with CMW 2. For the most common pouch cement brands, the LROI [17] reported highest cumulative revision risk at 10 years in hip procedures fixated with Simplex ABC Tobra (Stryker, Portage, USA) (CRR 6.6; CI 95%, range 5.5–7.8), and lowest risk of revision in Palacos MV+G (Heraeus Medical, Wehrheim, Germany) (CRR 3.6; CI 95%, range 2.9–4.4). In pre-packed vacuum mixing systems, Refobacin R was associated with highest revision risk (CRR 4.8; CI 95%, range 4.2–5.4) and Refobacin Plus with lowest risk (CRR 3.5; CI 95%, range 2.8–4.2).

When comparing knee replacements due to all causes Palacos R+G Pro (Heraeus Medical, Wehrheim, Germany) was associated with significantly lower revision risk (adjusted HR 0.86; CI 0.76–0.96; *p* < 0.0089) compared to Optipac Refobacin R (Zimmer Biomet, Warsaw, IN, USA) (HR 1 reference) after 10 years, while SmartSet GVH had an increased risk (HR 1.62, 95% CI 1.22–2.15; *p* = 0.0008). Analyzing cement types independently of mixing systems, SmartSet GVH showed an increased revision risk for all causes (HR 1.56, 95% CI 1.18–2.07; *p* = 0.0018) as well as all causes excluding infection (HR 1.68, 95% CI 1.17–2.4; *p* = 0.0046); however, Palacos R+G was associated with lower revision risk in all causes excluding infections (HR 0.87, 95% CI 0.78–0.97; *p* = 0.0136) and for prosthesis loosening (HR 0.81, 95% CI 0.66–0.99; *p* = 0.0362). The SAR concluded that prefilled mixing systems may be beneficial and that Palacos R+G irrespective of the cement mixing system may have lower revisions risk due to causes other than infections [39]. The LROI knee arthroplasty analyses [17] found that for most common pouch cement brands Simplex ABC EC (CRR 7.3; CI 6.5–8.0), Refobacin R (CRR 6.6; CI 6.1–7.1), and Refobacin Plus (CRR 6.5; CI 5.6–7.3) are associated with the highest risks of revision at 10 years. Whereas Simplex ABC Tobra (CRR 4.7; CI 4.0–5.3) and Palacos MV+G (CRR 5.3; CI 4.7–5.8) showed the lowest revision risks. Within the prepacked vacuum mixing systems, cumulative revision risk indicates better implant survival in Refobacin Plus (CRR 5.3; CI 4.9–5.7) than in Refobacin R (CRR 5.6; CI 5.3–6.0).

## Reduction of infection risk

Polymethyl methacrylate (PMMA) bone cement can also serve as a drug carrier for delivery of local antibiotics [36, 37]. If the PMMA cement contains only one antibiotic for use in primary procedures, it is referred to as single-loaded bone cement (SALBC). A dual-antibiotic loaded bone cement (DALBC) contains two different antibiotics and can be used for revision procedures or the treatment of high-risk patients [27].

**Fig. 6:**
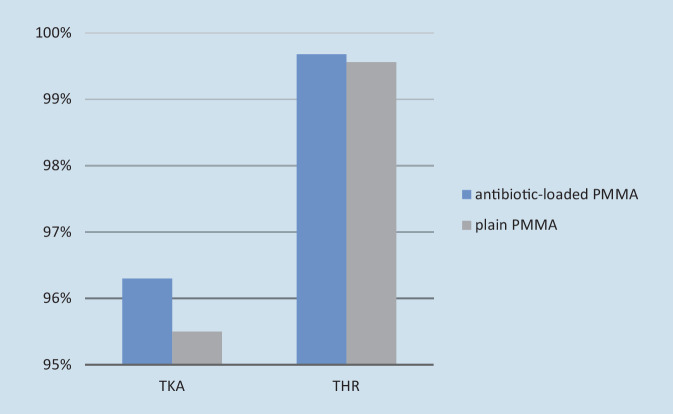
Survival rate after 10 years with PPI (periprosthetic joint infection) as the reason for revision. Graph according to Jameson et al. 2019 [31] and Leong et al. 2020 [40]. *TKA* total knee arthroplasty, *THA* total hip artroplasty

A comprehensive meta-analysis by Farhan-Alanie (370,000 hip and 670,000 knee arthroplasties) shows the protective effect of antibiotic-loaded bone cement (ALBC) against periprosthetic joint infection (PJI): the results are statistically significant for primary THAs (RR 0.6, 95% CI 0.56–0.77; *p* < 0.001) [23]. In the USA, the use of ALBC in primary procedures is often questioned, although Parvizi et al. [55] were already able to prove in a comprehensive meta-study in 2008 that ALBC can significantly reduce the risk of infection in primary arthroplasty. A further study of 15,972 primary TKAs in US veterans (4741 TKAs with plain PMMA cement and 11,231 TKAs with antibiotic-loaded PMMA cement) showed lower revision rates due to PJI in the patient group with ALBC [7]. Jameson et al. [31] observed an implant survival rate of 96.3% after 10 years for TKAs with ALBC compared to 95.5% with plain PMMA cement (Fig. 6). In addition, a lower risk of PJI (HR 0.79) and a simultaneous reduction in the risk of revision due to aseptic loosening or osteolysis was shown for THAs with ALBC compared to plain PMMA cement [40]. Kunutsor et al. [38] conducted a comprehensive review of the correlation between the fixation method (cemented, cementless, hybrid, reverse-hybrid) and the PJI risk of primary THAs. Within the first 6 months, the cementless THAs showed an increased risk of PJI compared to all cemented replacements (RR 0.75; 95% CI: 0.63–0.89). In contrast to anchoring with ALBC, antibiotic-free fixations were also associated with an increased PJI risk compared to cementless THAs [38]. The FNOF patients with multiple comorbidities have an increased PJI risk. The anchoring of hip stem implants with ALBC can therefore be recommended in the treatment of FNOF patients [48].

## Conclusion

- Based on international registry data, cemented TKAs show better survival rates compared to cementless TKAs.
- According to the register data, a cemented hip stem is advisable for older patients and is associated with a lower risk of revision compared to cementless fixation.
- Cemented hemiarthroplasty following a femoral neck fracture results in a lower risk of periprosthetic fractures and is a cost-effective treatment.
- Register data show no increased mortality with cemented hemiarthroplasty.
- The choice of bone cement brand influences the risk for revision.
- Register data and meta-analyses prove that antibiotic-loaded bone cement is the gold standard in infection prophylaxis.
